# Comparative Analysis of Dehydrins from Woody Plant Species

**DOI:** 10.3390/biom14030250

**Published:** 2024-02-20

**Authors:** Milan Karas, Dominika Vešelényiová, Eva Boszorádová, Peter Nemeček, Zuzana Gerši, Jana Moravčíková

**Affiliations:** 1 Institute of Biology and Biotechnology, Faculty of Natural Sciences, University of Ss. Cyril and Methodius in Trnava, Nám. J. Herdu 2, 917 01 Trnava, Slovakia; milannkaras@gmail.com (M.K.); dominika.veselenyiova@ucm.sk (D.V.); zuzana.gersi@ucm.sk (Z.G.); 2 Institute of Plant Genetics and Biotechnology, Plant Science and Biodiversity Center Slovak Academy of Sciences, Akademická 2, P.O. Box 39A, 950 07 Nitra, Slovakia; eva.boszoradova@savba.sk; 3 Institute of Chemistry and Environmental Sciences, Faculty of Natural Sciences, University of Ss. Cyril and Methodius in Trnava, Nám. J. Herdu 2, 917 01 Trnava, Slovakia; peter.nemecek@ucm.sk

**Keywords:** abiotic stress, dehydrins, dehydrin regulatory elements, phylogeny of dehydrins, shrub species, tree species, woody plants, vines

## Abstract

We conducted analyses on 253 protein sequences (Pfam00257) derived from 25 woody plant species, including trees, shrubs, and vines. Our goal was to gain insights into their architectural types, biochemical characteristics, and potential involvement in mitigating abiotic stresses, such as drought, cold, or salinity. The investigated protein sequences (253) comprised 221 angiosperms (85 trees/shrubs and 36 vines) and 32 gymnosperms. Our sequence analyses revealed the presence of seven architectural types: K_n_, K_n_S, SK_n_, Y_n_K_n_, Y_n_SK_n_, FSK_n_, and F_n_K_n_. The FSK_n_ type predominated in tree and shrub dehydrins of both gymnosperms and angiosperms, while the YnSKn type was more prevalent in vine dehydrins. The Y_n_SK_n_ and Y_n_K_n_ types were absent in gymnosperms. Gymnosperm dehydrins exhibited a shift towards more negative GRAVY scores and Fold Indexes. Additionally, they demonstrated a higher Lys content and lower His content. By analyzing promoter sequences in the angiosperm species, including trees, shrubs, and vines, we found that these dehydrins are induced by the ABA-dependent and light-responsive pathways. The presence of stress- and hormone-related cis-elements suggests a protective effect against dehydration, cold, or salinity. These findings could serve as a foundation for future studies on woody dehydrins, especially in the context of biotechnological applications.

## 1. Introduction

Plants are constantly exposed to various types of stress throughout their life, which can have detrimental effects on plant physiology, metabolism, and overall performance, impacting plant growth, reproduction, and survival. Abiotic stress causes cell damage, triggers osmotic stress and, subsequently, oxidative stress, which in turn leads to the activation of a plant’s defense mechanism. A part of the plant stress response is the modulation of cellular metabolism and production of a wide range of proteins including dehydrins. Dehydrins (PF00257) are also known as group 2 “late embryogenesis abundant (LEA) proteins” [1], which were originally associated mainly with the later stages of plant embryo development; however, their role in conditions of a/biotic stress has also been suggested [2]. Dehydrins are proteins that are highly hydrophilic and rich in glycine and lysine. They can retain a large amount of water and thus protect other biomolecules and cell membrane structures from water loss [3]. As intrinsically disordered proteins [4], dehydrins can flexibly respond to environmental stimuli and perform molecular functions associated with their roles in mitigating water-related stress [5]. Research on dehydrins has been increasing in recent years due to their potential applications in crop improvement and management of environmental stress.

Dehydrins are characterized by their highly conserved sequences called K, S, and Y segments. Based on the occurrence of the conserved fragments, their frequency, and mutual combination, dehydrins can be divided into several subgroups: K_n_, SK_n_, K_n_S, Y_n_K_n_, and Y_n_SK_n_ [6]. The K segment is characteristic of all dehydrins and is considered a well-conserved sequence in dehydrins. The segment consists of lysine-rich residues (EKKGIMDKIKEKLPG) [7]. Sequence analyses revealed variability in the K segment described as (XKXGXX(D/E)KIK(D/E)KXPG), where X denotes any amino acid [5]. The K segment can bind to phospholipid membrane surfaces, create amphipathic α-helices, and stabilize membranes by the shielding effect [8]. The Y segment (DEYGNP) is initially named according to the tyrosine residue in the middle of the motif [7]. The position of tyrosine is more variable (D(D/E)(Y/H/F)GNPX), where X represents a hydrophobic amino acid [5]. Analyses showed sequence similarity between the Y segment and nucleotide binding domains of chaperones [7]. The S segment [LHR(S/T)GS_4–6_(S/D/E)(D/E)_3_] usually consists of 4–6 serine residues. The S segment often undergoes phosphorylation, which leads to the binding of calcium ions [9] and a translocation of dehydrins to the nucleus [10]. In addition, some dehydrins also have a motif so-called the F segment [(EXXDRGXFDFX(*G*/*K*)], named for the presence of two phenylalanine residues [11].

Dehydrins also contain poorly conserved regions named Φ segments that are enriched with glycine and polar amino acid residues, especially threonine [12]. Φ segments have a helix structure with the ability to bind a large amount of water through peptide bonds with water molecules and, in most cases, lie between K segments [13]. Most studies focusing on dehydrins have been performed on annual plants, especially on *Arabidopsis* as a model plant. Genome analysis of conserved dehydrin motifs in vascular plants (including some woody plants) showed that some architectural types may be expressed during certain abiotic stress, while others may be involved in all types of abiotic stress (drought, cold, salinity) [6]. However, a comparable study focusing solely on herbaceous plants has not yet been conducted. Woody plants (trees, shrubs, vines) are less convenient experimental objects due to their long life cycle and large size. The duration of this life cycle varies significantly among species, ranging from a few years for some fast-growing species to several centuries for long-lived species. The long biological cycle of woody plants makes them more vulnerable to environmental stress. Maintaining the water balance is important for their growth and development, as water transport and xylem conductivity influence photosynthesis and carbon dioxide fixation [14]. The main role of dehydrins is to protect the developing seeds from cellular damage caused by dehydration [15]. Not surprisingly, woody dehydrins have been studied mainly in relation to their role during drought and cold stress. In this study, we performed analyses of 253 dehydrin sequences to identify their architectural types, biochemical properties, and their role in response to drought, cold, and salt stress. The investigated protein sequences comprised 221 angiosperms (85 trees/shrubs and 36 vines) and 32 gymnosperms. Understanding the roles of dehydrins in the tolerance of plants to abiotic stress can lead to the development of stress-tolerant crops and strategies for mitigating the impact of stresses on agriculture and natural ecosystems.

## 2. Materials and Methods

### 2.1. Dehydrin Sequences and Motif Search

The protein sequences (Pfam00257) were obtained from Uniprot database. Duplicate sequences and fragments were removed before the analyses. Using the MEME software 5.5.2 (https://meme-suite.org, accessed on 4 April 2023), we filtered sequences containing at least one K segment with the conserved core section and, eventually, the Y, S, and F segments. The results were visualized using the LOGO format and as a position-specific probability matrix [16]. The architectural types of dehydrins were determined based on the presence and number of the K, S, Y, and F segments.

The dataset of protein sequences (253) encompassed dehydrins from trees, shrubs, and vines of 25 distinct species, included 221 angiosperm (185 trees/shrubs and 36 vines) and 32 gymnosperm (trees) dehydrins (Table S1).

### 2.2. Calculation of Biochemical Properties

The theoretical biochemical properties molecular weight (Mw), isoelectric point (pI), GRAVY (grand average of hydropathicity) score, and histidine and lysine percentages were calculated using the ProtParam tool (https://web.expasy.org/protparam/, accessed on 9 May 2023). The fold index of proteins was calculated using FoldIndex© software version 1.68 (https://fold.proteopedia.org/cgi-bin/findex#about, accessed on 19 May 2023). Data were visualized using box plots. The non-parametric test (Games–Howell) was performed to evaluate differences between the gymnosperms (trees), angiosperms (trees/shrubs), and angiosperms (vines) for each studied variable (MW, pl, GRAVY, fold index, Lys% and His%).

### 2.3. Phylogenetic Tree Construction

The protein sequences were aligned using MAFFT software version 7 (accessed on 21 June 2023) [17] with default settings for amino acid sequences. The multiple alignment was used as an input set to calculate the evolutionary relationships between dehydrin sequences. Dehydrins with atypical structures were not included. The phylogenetic tree was calculated from 247 sequences using IQ-Tree software, version 2 [18], with the JTT + F + G4 model [19]. The bootstrap value was set to 1000. The phylogenetic tree was visualized and graphically edited using the online program ITOL version 5 (https://itol.embl.de, accessed on 15 November 2023).

### 2.4. Obtaining of Promoter Sequences and Analyses of Cis Regulatory Sequences

The 2 kb upstream DNA sequences of dehydrin genes were acquired from the Phytozome 13 database (https://phytozome-next.jgi.doe.gov/, accessed on 11 October 2023) [20]. The dataset is comprised of 34 promoter sequences of angiosperm dehydrins from 8 species (*Citrus sinensis*, *Gossypium raimondii*, *Gossypium tomentosum*, *Gossypium mustelinum*, *Malus domestica*, *Populus trichocarpa*, *Populus deltoides*, and *Vitis vinifera*). The sequences were submitted to the PlantCare online tool (https://bioinformatics.psb.ugent.be/webtools/plantcare/html/, accessed on 6 November 2023) to identify cis-acting regulatory elements [21].

## 3. Results

### 3.1. Presence of K, Y, S, and F Segments in Dehydrins

**Figure 1:**
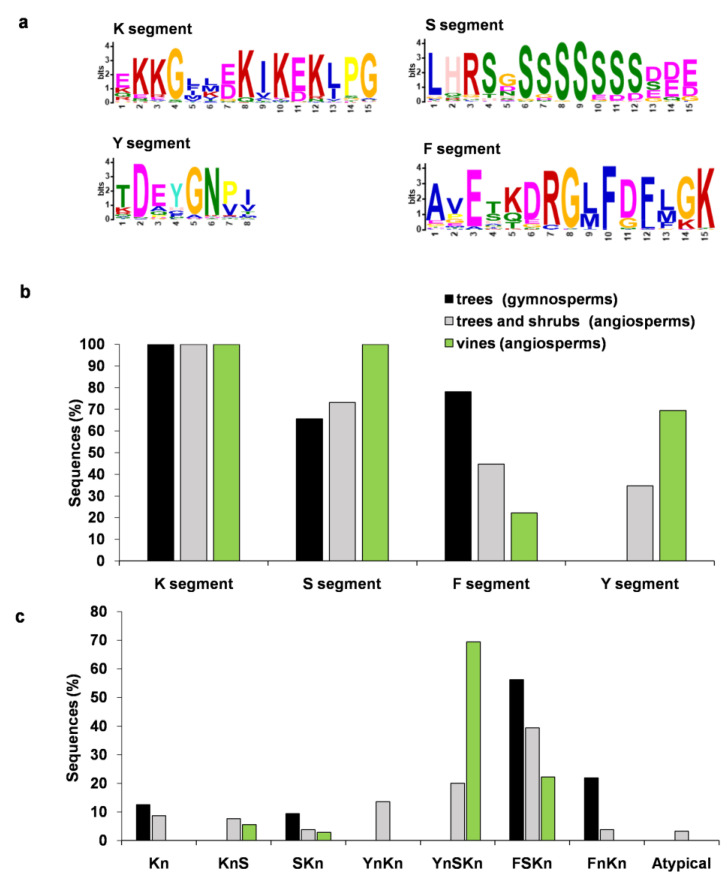
MEME LOGO of K, Y, S, and F segments in dehydrins of woody plants (**a**). Distribution of dehydrin sequences based on the presence of the K, S, F, and Y segments (**b**). Distribution of dehydrin sequences based on their structural types (**c**).

The protein sequences of 25 plant species (trees, shrubs, and vines) named as dehydrins (PF00257) were acquired from the Uniprot database. As the K segment is obligatory for dehydrins [7], we first searched for the sequences containing at least one K segment with the conserved core sections. Using the MEME program, we filtered 253 dehydrin sequences (Figure 1a, Table S1). These sequences covered 221 angiosperm (trees, shrubs, and vines) and 32 gymnosperm (trees) dehydrins. Since some plant species are listed as small trees or shrubs (e.g., *Rhaphiolepis bibas* or *Coffea canephora*), we decided to divide the angiosperm sequences into two main groups. One group (185) comprised tree and shrub dehydrins, while the second group (36) consisted of vine dehydrins. This division allowed for us to analyze the sequences separately, considering their distinct growth characteristics and potential differences in stress responses. Then, the dataset was analyzed to find the Y, S, and F segments. The LOGO representation of the K, Y, S, and F segments and the position specific probability matrixes (PSPM) are given in Figure 1a and Tables S2–S5 (respectively).

In the K segment, the Lys residue was situated with a high frequency at positions two and three (81%), eight and ten (90%) and twelve (86%) (Table S2). In some cases, the Ile residue in the most conservative part of the K segment (Lys-Ile-Lys-Glu) [7] was replaced by another hydrophobic and non-aromatic amino acid, Val (12%). The amino acids Gly and Pro were highly conserved at positions four and fifteen (>91%) and fourteen (84%), respectively. The remaining positions of amino acid residues were variable. The number of the K segments mostly varied between one and nine. In some dehydrins, the K segment was flanked by His residues (Table 1 and Table S1).

The MEME analyses revealed that 34.4% of the dehydrins in this study contained the Y segment. We did not find the Y segment in the sequences of gymnosperm dehydrins (Figure 1b). The LOGO presentation of the Y segment consisted of the conserved residues at positions between two and six. The most conserved amino acid residues were Asp (96%), Gly (91%) and Asn (92%). In the middle of the motif (positions three and four), Glu and Tyr residues were found with the frequencies of 65% and 62% (respectively) (Table S3). The Y segment was located at the N-terminus and mostly in one or two copies. There was also a small group (five) of the Y-containing dehydrins with three, six or seven copies, e.g., dehydrins of *Prunus armeniaca* (A0A6J5VHL9) or *Juglans regia* (A0A6P9EPF4 and A0A6P9EQ41), respectively.

The S segment was found in 74.3% of analyzed sequences in both angiosperm (trees/shrubs 73.2%, vines 100%) and gymnosperm (trees, 65.6%) dehydrins (Figure 1b). The MEME LOGO of the S segment showed that it most likely consists of seven consecutive Ser residues (at positions 6–12). The Ser tract is usually formed of 4–8 Ser residues [22]. At the N-terminus, there was found a well-conserved motif consisting of the amino acids Leu (87%), His (81%), and Arg (86%), followed by Ser (80%) (Table S4). At the C-terminus, the Ser tract is followed by Asp/Glu. In some dehydrin sequences, the S segment was accompanied by a poly lysine motif (KKKKKEKK) (Table 1 and Table S1).

More than 44% of analyzed dehydrins harbored the recently discovered F segment [11]. The F segment was detected in both gymnosperm (trees, 78.1%) and angiosperm (trees/shrubs 44.7%, vines 22.2%) dehydrins (Figure 1b). The most conserved amino acids in the LOGO of the F segment were Gly (97%), Lyz (97%), Glu (94%), Phe (98% and 81%), and Arg (92%) at positions eight, fifteen, three, ten and twelve, and seven (respectively) (Table S5). The F segment at one copy was commonly found at the N-terminus. An exception is a dehydrin from *Quercus lobata* (A0A7N2KTG4) with two copies of the F motif.

The architecture of the analyzed dehydrins falls into seven types: K_n_, K_n_S, SK_n_, Y_n_K_n_, Y_n_SK_n_, FSK_n_, and F_n_K_n_. The most prevalent types were Y_n_SK_n_ and FSK_n_ (Figure 1c). In gymnosperms, the FSK_n_ (56.3%) and F_n_K_n_ (21.0%) types were the most represented. In angiosperms, the FSK_n_ type predominated in trees and shrubs (39.5%), while the Y_n_SK_n_ type was more prevalent in vines (69.4%). The K_n_, Y_n_K_n_, and F_n_K_n_ types were not observed in vine dehydrins. In addition, there was a group of (six) angiosperm dehydrins, found exclusively in tree and shrub species, that did not follow the above-mentioned architecture. This group includes, for example, the dehydrins with the architecture YSK_2_(KS)SK_3_Y_2_SK_3_ from *Malus domestica* (A0A498KQG3) or Y_2_(KY_3_)_3_Y_2_K_2_ from *Juglans regia* (A0A833UHG8) (Figure S1).

**Figure 2:**
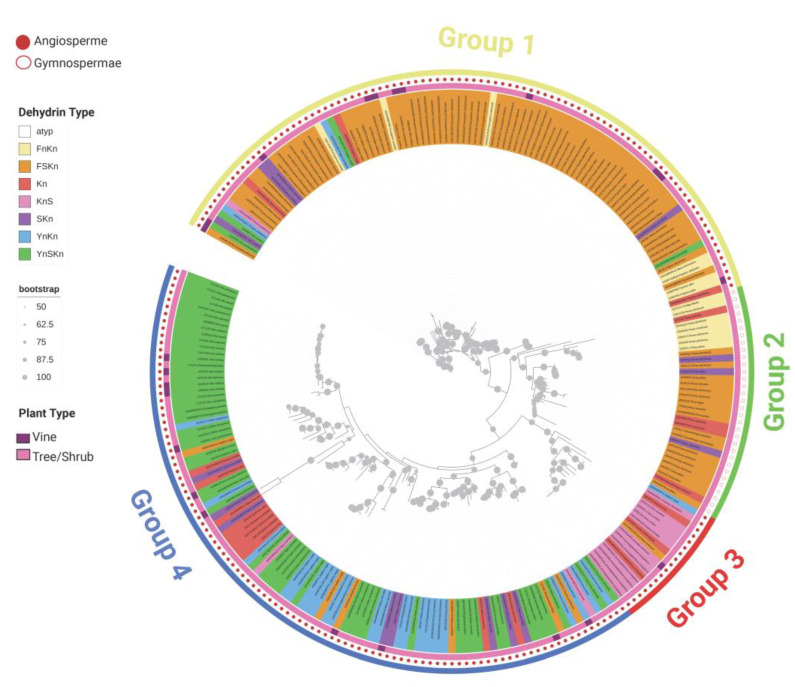
Phylogenetic tree analysis of dehydrin proteins. The tree was built from 247 dehydrin amino acid sequences (Table S1). Sequences were clustered into 4 distinct groups (group 1 (*n* = 94, FSK_n_ 84.0%), group 2 (*n* = 32, FSK_n_ 56.3%, F_n_K_n_ 21.9%), group 3 (*n* = 17, K_n_S 88.2%), group 4 (*n* = 104, Y_n_SK_n_ 57.7%, Y_n_K_n_ 24.0%)). The bootstrap value was set to 1000. The phylogenetic tree was visualized and graphically edited using the online program ITOL version 5.

The phylogenetic tree was constructed with 247 out of 253 dehydrin sequences (Figure 2). The dehydrins with atypical structures were excluded from the analyses. The tree could be roughly divided into four groups. The first group (*n* = 94) mainly consisted of the type FSK_n_ (84.0%) and did not include the K_n_ and Y_n_K_n_ types. The second group (*n* = 32) comprised the types FSK_n_ (56.3%) and F_n_K_n_ (21.9%). None of the sequences in this group contained the Y segment. The third group (*n* = 17) primarily included the K_n_S (88.2%) type. The fourth group (*n* = 104) encompassed the types Y_n_SK_n_ (57.7%) and Y_n_K_n_ (24.0%). All gymnosperm dehydrins were classified within group two. Angiosperm dehydrins were distributed among the groups one, three, and four.

### 3.2. Analysis of Architecture, Biochemical Characteristics, and Protective Properties of Dehydrins in Woody Plants

In general, the biochemical properties of dehydrins depend, to a large extent, on their architecture [6]. We analyzed and compared the molecular weight (Mw), isoelectric point (pI), net hydrophobicity (GRAVY score), and propensity to fold (fold index) in dehydrins from gymnosperms (trees) and angiosperms (trees/shrubs and vines).

**Figure 3:**
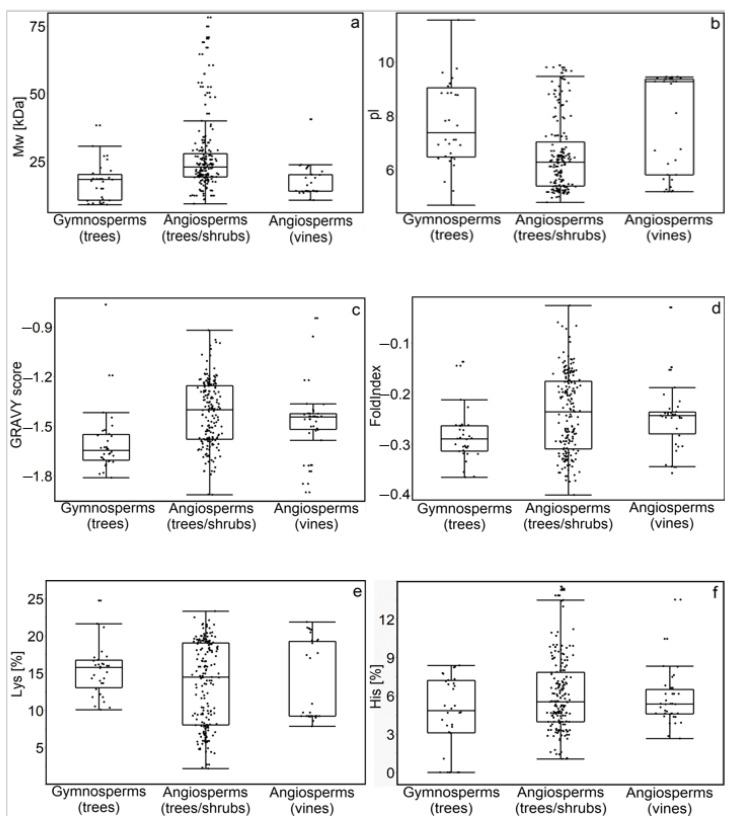
Biochemical properties of dehydrins in woody plants categorized by the gymnosperm (trees) and angiosperm (trees/shrubs and vines) dehydrins. Molecular weight (Mw) (**a**), isoelectric point (pI) (**b**), GRAVY score (**c**), fold index (**d**), Lys content (**e**), His content (**f**).

The molecular weight was centered from 17 kDa to 25.9 kDa (angiosperms; vines and trees/shrubs, respectively) (Figure 3a). The statistical analyses revealed significant differences (at *p* < 0.05) in the molecular weights of angiosperm (trees/shrubs) dehydrins when compared to gymnosperm (trees) dehydrins. However, no significant differences were observed between gymnosperm (trees) and angiosperm (vines) dehydrins.

Analyses of the isoelectric point (pI) (Figure 3b) revealed a predominance of acidic pI (centered at approximately 6.5) for the angiosperm (trees/shrubs) dehydrins, while the majority of gymnosperm (trees) and angiosperm (vines) dehydrins exhibited a pI of approximately 7.7 and 7.9, respectively. Similarly to the molecular weight, the statistical analyses revealed significant differences (at *p* < 0.05) in the isoelectric point (pI) between angiosperm (trees/shrubs) and both gymnosperm (trees) and angiosperm (vines) dehydrins.

GRAVY analyses (Figure 3c) revealed that all dehydrins had a negative score, indicating their hydrophilicity. For the angiosperm (trees/shrubs and vines) dehydrins, the GRAVY scores were centered at approximately −1.42 and −1.47 (respectively), whereas for the gymnosperm (trees) dehydrins, this was approximately −1.6.

The negative scores of the fold index (Figure 3d) indicate that dehydrins in both gymnosperms and angiosperms are intrinsically disordered. Furthermore, the fold index scores correlate with the GRAVY scores, suggesting a connection between disorder and the presence of hydrophilic residues [6]. The statistical analyses revealed significant differences (at *p* < 0.05) in the fold index and GRAVY score between gymnosperm (trees) and angiosperm (trees/shrubs and vines) dehydrins.

For the angiosperm (trees/shrubs and vines) dehydrins, the representation of Lys residues was concentrated at approximately 13%, while for the gymnosperm (trees) dehydrins, it was approximately 15.4% (Figure 3e). The highest (approximately 6.1% and 5.8%) percentage of His residues was found in the angiosperm (trees/shrubs and vines) dehydrins, respectively. For the gymnosperm (trees) dehydrins, the representation of His residues was approximately 4.6%. The statistical analyses revealed significant differences (at *p* < 0.05) in the percentage of Lys and His residues between gymnosperm and angiosperm dehydrins (Figure 3f).

Out of the 253 studied dehydrins, 53 (3 gymnosperm and 50 angiosperm dehydrins) were experimentally identified as responsive to drought, cold, salt, or metal stress (Tables S1 and S6). Some dehydrins were upregulated in response to multiple stress types, including drought/cold, drought/salinity, or cold/salinity. For example, the dehydrin (Y_n_SK_n_, Q3ZNL5) from *Vitis riparia* was induced by drought and cold [23], while the K_n_S dehydrin (A0A346RSN0) from *Ammopiptanthus mongolicus* was induced by cold and salinity [24]. In addition, the dehydrins from *Populus alba* (FSK_n_, Q0MRE0) [25] and *Actinidia deliciosa* (Y_n_SK_n_, A0A8K1V8R6) [26] (respectively) were induced by drought, cold, and salinity.

### 3.3. Regulation of Dehydrins

**Figure 4:**
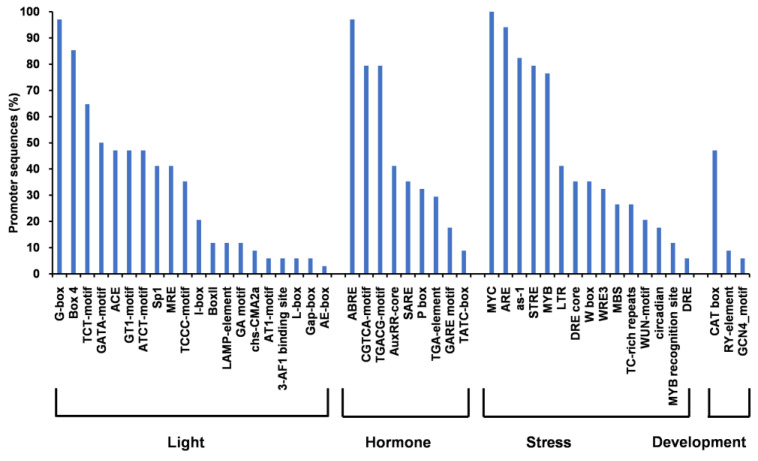
The frequency of occurrence of cis-acting regulatory elements in the promoter sequences of dehydrin genes with the *Y*-axis representing the percentage of promoter sequences containing the corresponding cis element at least once.

Furthermore, we analyzed the promoter sequences of 34 dehydrins [FSK_n_ (16), Y_n_SK_n_ (9), F_n_K_n_ (3), K_n_ (4) SK_n_ (2)] obtained from the Phytozome database [20] (Table S1). The dataset contained promoters of angiosperm dehydrin genes [trees/shrubs (25), and vines (9)]. The PlantCare database predicted potential cis elements associated with plant growth, development, hormones, and a/biotic stress (Figure 4, Table S7). All analyzed sequences contained multiple cis elements associated with light responsiveness (Table S7). The most frequently represented were the G-box and Box 4. Motifs involved in hormone regulation, such as abscisic acid (ABRE) and methyl jasmonate (MeJA)-responsive elements, were found in 97% and 79% of the promoters, respectively. Additionally, cis elements related to other hormones included auxin-responsive elements (AuxRR-core and TGA element), the salicylic acid-responsive element (SARE), and gibberellin-responsive elements (P-box, TATC box, and GARE motif). The promoter sequences also contained stress-responsive cis elements, including MYC (stress-responsive), ARE (anaerobic induction), as-1 (oxidative stress-responsive), MYB (stress-responsive), LTR (low temperature-responsive), DRE core (a coupling element of ABA), W box (a binding site for WRKY TFs), WRE3 (wounding and pathogen responsiveness), MYB transcription factor binding site (drought-responsive), TC-rich repeats (associated with defense and stress response), MYB recognition site (responsive to water stress), WUN motif (responsive to wounding), DRE (responsive to drought, low temperature, and salt), and circadian (under circadian control). The presence of cis elements, such as CAT box, RY motif, and GCN4_motif, suggests that some dehydrins may be involved in seed maturation, seed dormancy, and/or meristem-specific activation.

## 4. Discussion

Dehydrins are a group of intrinsically disordered proteins that play a crucial role in enhancing the plant’s ability to survive and adapt to challenging environments, making them an essential component of plant stress tolerance mechanisms. These proteins are characterized by their conserved regions known as K, S, Y, and F segments. Our study of 253 protein sequences, including gymnosperms (trees) and angiosperms (trees/shrubs and vines), revealed the K motif with slight variations in the conserved residues at the N-terminal (Glu-Lys-Lys and Lys-Ile-Lys-Glu) and at the C-terminal of the segment (Lys-Leu-Pro-Gly). Due to properties such as hydrophobicity and positive charge, the K segment plays a key role in the function of dehydrins. The core sequence (Lys-Ile-Lys-Glu) is crucial for cryoprotection, as any changes in this sequence that lead to the loss of hydrophobicity cause a significant reduction in their cryoprotective activity [27,28]. Moreover, the positive charge facilitates the binding of dehydrins to negatively charged intracellular components, such as cell membranes, providing protection under low-temperature conditions [28,29,30]. We have found some dehydrins with the K segments flanked by His-His residues (Table 1 and Table S1), a configuration believed to enhance interactions between the K segment and membranes in a pH-dependent manner [31]. Given that the global charge can serve as a crucial secondary regulator of membrane association [32], it is plausible that the positively charged dehydrins from *Actinidia deliciosa* (Y_n_SK_n_, A0A8K1V8R6) or *Malus baccata* (Y_n_K_n_, A0A540MKP4) may exhibit a high affinity to negatively charged vesicles. Analysis of the dehydrin (A0A8K1V8R6) of *A. deliciosa* [26] demonstrated its inducibility under drought, cold, and salt stress.

The S segment was found in dehydrins of both gymnosperms and angiosperms. The S segment was formed from the Ser tract consisting of seven Ser residues and highly conserved amino acid residues Leu-His-Arg (at the beginning of the N-terminus of the S segment). This segment is thought to harbor a potential phosphorylation site for kinases, such as casein kinase II and SNF1-related kinases [33]. Phosphorylation potentially contributes to nuclear targeting of some dehydrins and promotes their binding to calcium ions [9,33]. Nuclear localization of dehydrins indicates their protective ability in the nucleus. One potential function is to protect DNA from oxidative stress induced by attacks from reactive oxygen species (ROS). For instance, the K_n_S dehydrin (CuCOR15) from *Citrus unshiu* (Q50LG8) has been shown to have the ability to interact with DNA in the presence of zinc ions [34]. Similarly, the Y_n_SK_n_ dehydrin VrDHN1 from *Vitis riparia* (Q3ZNL5) can protect DNA from damage caused by hydrogen peroxide through nonspecific binding to DNA [35]. Hara et al. [36] demonstrated ROS scavenging activities of the K_n_S dehydrin CuCOR19 from *Citrus unshiu* (Q9ZR21). An overexpression of the dehydrin CuCOR19 enhanced cold tolerance in transgenic tobacco plants by reducing lipid peroxidation. Some studies have pointed to the role of dehydrins in the prevention of ROS formation by sequestering free metal ions released during abiotic stress [8]. The K_n_S dehydrin CuCOR15 from *Citrus unshiu* (Q50LG8) exhibited the ability to bind metals through the sequence HKGEHHSGDHH [37]. We have found a similar motif in the cold-responsive K_n_S dehydrin COR15 from *Citrus paradisi* (Q93XL8) (Table S1). The ability to bind metal ions through the specific (HH-3X-HH) sequence might be a mechanism by which dehydrins alleviate toxicity of free metal ions released during stress.

In some dehydrin sequences of angiosperms (trees/shrubs) (Table 1), the S segment was accompanied by a poly K motif (KKKKKEKK). The poly K motif can act as a polar zipper and promotes interaction between protein complexes [10,34,38].

The Y segment was found only in the dehydrins of angiosperms. This suggests that the Y segment-containing dehydrins of woody plants arose after the divergence of seed plants into gymnosperms and angiosperms. A total of 66.7% of vine dehydrins contained the Y segment, while in trees and shrubs, the Y segment occurred with a frequency of 26.5% (Figure 1). The initial assumption that the Y segment might act as a nucleotide binging domain [12] was not experimentally demonstrated in vines [35]. Trees and shrubs are part of the early plant evolution. Vines, on the other hand, represent a later adaptation that evolved as a strategy for climbing and reaching sunlight in dense vegetation. The Y segment-containing dehydrins might contribute to the improved adaptation of angiosperms to environmental conditions.

The recently discovered F segment has been identified in both gymnosperm and angiosperm dehydrins, including vines. Among gymnosperm dehydrins, 78.1% featured the F segment, while in trees and shrubs of angiosperms, the F segment occurred with a frequency of 48.2%. In vine dehydrins, the F segment was less presented (22.3%). Since gymnosperms predate angiosperms, the F segment might be evolutionarily older than the Y segment. The higher prevalence of the F segment in gymnosperm dehydrins might be linked to its protective function in “naked” seeds, where the absence of an ovary wall might require additional protection, while in coated seeds, the significance of this protective role likely gradually diminishes. The core of the F segment (Leu-Phe-Asp-Phe-Leu) is highly conserved, and its hydrophobicity was predicted necessary for enzyme cryoprotective activities [39].

Various combinations of the K, S, Y, and F segments ensure dehydrins a high degree of diversity, enabling them to perform tasks not only during plant growth and development but also under stress conditions. We identified seven architectural types: K_n_, K_n_S, SK_n_, Y_n_K_n_, Y_n_SK_n_, FSK_n_, and F_n_K_n_. Angiosperms included all structural types of dehydrins, while gymnosperms lack the types Y_n_K_n_ and Y_n_SK_n_. The FSK_n_ type was most common in trees and shrubs of both gymnosperm and angiosperm dehydrins, whereas the Y_n_SK_n_ type was most prevalent in vine dehydrins. The phylogenetic tree categorized dehydrins into four distinct groups. Angiosperm dehydrins were found in the groups one, three, and four, while gymnosperm dehydrins were placed within group two. This can be associated with the lower variability of architectural types in gymnosperm dehydrins. The lower variability of the architectural type may also result from a smaller sample size (32 gymnosperm dehydrins) compared to angiosperms. All gymnosperm dehydrins lacked the Y segment with the predominant architectural types being FSK_n_ and F_n_K_n_, accounting for 78.1%. Groups one and two predominantly encompassed FSK_n_ dehydrins. The majority of dehydrins in group three were of the K_n_S type. Group four consisted of dehydrins belonging to the Y_n_K_n_ and Y_n_SK_n_ types. Vine dehydrins were distributed among groups one and four.

The presence of different architectural types of dehydrins within a single species suggests that these could have arisen during genome evolution [40], whereby dehydrins acquired new functions. For example, *Prunus armeniaca* exhibits at least three structural types of dehydrins (FSK_n_, Y_n_SK_n_, and Y_n_K_n_). It is conceivable that, while the FSK_n_ type might have originated from ancestral dehydrins of spermatophytes, additional dehydrin structures may have arisen through duplication events combined with the loss of the F and S segments and the acquisition of the Y segment.

Among the primary environmental factors impacting various physiological and biochemical processes in woody plants, water scarcity and low temperature exert significant influences. All dehydrins exhibit hydrophilicity and are intrinsically disordered proteins, as indicated by their negative GRAVY scores and fold indexes (Figure 3). When compared to angiosperm (trees/shrubs and vines) dehydrins, gymnosperm (trees) dehydrins demonstrate a shift towards more negative GRAVY scores and fold indexes. Moreover, gymnosperm dehydrins show a higher content of Lys and lower content of His residues. This may be related to the role of dehydrins in the safeguarding of plant cells in gymnosperms from the detrimental effects of low temperatures.

The accumulation of dehydrins is closely related to seed viability, especially as the moisture content drops to 10% during maturation [15]. The last stage of embryogenesis involves the acquisition of desiccation tolerance, allowing seeds to withstand drying without losing viability. Dehydrins contribute to desiccation tolerance by stabilizing cellular structures and protecting proteins and membranes from damage caused by water loss. Recalcitrance is primarily associated with tropical plants that thrive in areas where frequent rainfall and high humidity prevail, and their seeds do not have to endure winter conditions. Unlike orthodox seeds, recalcitrant seeds cannot withstand desiccation; they lose viability if they dry out. The extent of their dehydration tolerance can vary depending on the plant species [41]. For example, dehydrins (types K_n,_ Q5UVI0; and Y_n_SK_n,_ Q5UVI2) have been identified in “atypical” recalcitrant seeds of *Quercus robur* [41,42], suggesting a probable gradual adaptation of oak seeds to environmental conditions. While seeds are multicellular structures, pollen grains are small, consisting of either two or three cells. Orthodox pollen is typically dispersed as bicellular pollen in a somewhat dehydrated state, while recalcitrant pollen with its higher water content (tricellular state) is advantageous in humid environments [43]. The accumulation of dehydrins is one of the factors associated with the desiccation tolerance of pollen [44].

Drought stress is not solely a consequence of inadequate water availability; it can also result from salinity, cold, or frost stress, all of which contribute to reduced water content within plant cells. Some dehydrins of woody species show inducibility in response to multiple types of stress [e.g., the FSK_n_ dehydrin of *Populus alba* (Q0MRE0) and Y_n_SK_n_ dehydrin of *Actinidia deliciosa* (A0A8K1V8R6)]. Moreover, the expression of individual dehydrins within species may vary depending on the environmental conditions. For instance, analyses of the dehydrin gene family in *Malus domestica* showed that the expression of some dehydrins [e.g., Y_n_SK_n_ MdDH4 (J9PZB0) and Y_n_K_n_ MdDH6 (J9Q0P7)] was significantly upregulated by drought/cold, while the expression of other dehydrins [e.g., the Y_n_SK_n_ MdDH1 (J9Q0P4)] was enhanced only by drought stress [45].

In general, the expression of stress-responsive dehydrins is triggered by signaling pathways involving ABA, MAPK, and Ca^2+^ ions [3] with Ca^2+^ serving as a bridge that links responses to drought, salinity, and cold stress. The analysis of promoter sequences in angiosperms, including trees, shrubs, and vines, reveals that dehydrins are induced by ABA and ABA-responsive cis elements with their expression being modulated by light (see Figure 4). The presence of cis elements such as LTR and DRE suggests that certain dehydrins may be induced through both ABA-dependent and ABA-independent stress-signaling pathways. However, these pathways are not mutually exclusive, and crosstalk between ABA-dependent and ABA-independent pathways has been observed [3]. The cis elements linked to ABA-dependent pathways were found across studied architectural types (FSK_n_, Y_n_SK_n_, F_n_K_n_, K_n_, SK_n_) and woody plant species, indicating conserved regulation of dehydrins, as previously described by [46]. The stress- and hormone-related cis elements identified in the promoters indicate a protective effect against dehydration, cold, or salinity. Conversely, cis elements associated with cellular development are less prevalent. They are involved in endosperm expression, seed-specific regulation, and/or meristem-specific activation.

## 5. Conclusions

This report describes the sequence analysis of dehydrins in woody plants, encompassing gymnosperms (trees) and angiosperms (trees/shrubs and vines). We identified seven architectural types: K_n_, K_n_S, SK_n_, Y_n_K_n_, Y_n_SK_n_, FSK_n_, and F_n_K_n_. Among these, the types FSK_n_ F_n_K_n,_ Y_n_K_n_, and Y_n_SK_n_ were the most represented. In gymnosperms (trees), the majority of dehydrins belonged to the FSK_n_ and F_n_K_n_ types, whereas in angiosperms (trees/shrubs), these types were less prevalent. Among vine dehydrins, the most represented types were Y_n_SK_n_ and FSK_n_. All gymnosperms lacked the types Y_n_K_n_ and Y_n_SK_n_, pointing to diverse adaptation strategies between gymnosperms and angiosperms in response to environmental conditions. Trees and shrubs are part of the early plant evolution. Vines, on the other hand, represent a later adaptation to the environment. Through phylogenetic analysis, we categorized dehydrins into four distinct groups. Dehydrins of gymnosperms demonstrate a shift towards more negative GRAVY scores and fold indexes. Moreover, they show a higher content of Lys and lower content of His residues. This may be related to the role of dehydrins in the safeguarding of plant cells in gymnosperms from the detrimental effects of low temperatures. The analysis of promoter sequences in angiosperm species, including trees, shrubs, and vines, shows that these dehydrins are induced by ABA-dependent and light-responsive pathways. The presence of stress- and hormone-related cis elements indicate a protective effect against dehydration, cold, or salinity. The results expand the knowledge regarding the structure and role of dehydrins in woody plants under abiotic stress conditions. The data obtained can serve as a source for conducting broader functional analyses of dehydrins, aiming to enhance our understanding of the functions these proteins serve in the abiotic stress response of woody plants.

## Figures and Tables

**Table 1:** Occurrence of the rare motifs in woody dehydrins according to the architectural types and species.

	K_n_	K_n_S	SK_n_	Y_n_K_n_	Y_n_SK_n_	F_n_K_n_	FSK_n_	Atypical
Motif	Ang(T,S/V) //Gym(T) [%]	Ang(T,S/V) //Gym(T) [%]	Ang(T,S/V) //Gym(T) [%]	Ang(T,S/V) //Gym(T) [%]	Ang(T,S/V) //Gym(T) [%]	Ang(T,S/V) //Gym(T) [%]	Ang(T,S/V) //Gym(T) [%]	Ang(T,S/V) //Gym(T) [%]
HH-K- seg-HH	-	-	-	8.0/0//0	1.6/3.3//0	-	-	-
Poly K motif	-	31.3/0//0	-	-	-	-	36.0/0//0	-
His-rich motifs:								
H-H ^a^	-	18.0/0//0	36.4/0//0	12.0/0//0	3.3/0//0	-	9.0/1.0//0	1.7/0//0
HH-X_3_-HH ^b^	-	1.7/0//0	-	-	-	-	-	-

Ang—angiosperms, T—trees, S—shrubs, V—vines; Gym—gymnosperms; poly K motif—KKKKKEKK. ^a^ As His-His (HH) rich motif were considered the sequences containing four or more HH residues. ^b^ HH-X_3_-HH motif denotes HH-Ser-Gly-Asp-HH.

## Data Availability

All data generated during this study are included in this published article (and its Supplementary Materials).

## Supplementary Materials

The following supporting information can be downloaded at: https://www.mdpi.com/article/10.3390/biom14030250/s1, Table S1: List of dehydrins used in analyses, Table S2: Position-specific probability matrix of the K segment, Table S3: Position-specific probability matrix of the Y segment, Table S4: Position-specific probability matrix of the S segment, Table S5: Position-specific probability matrix of the F segment, Table S6: Distribution in architectural types of dehydrins within plant species and their inducibility under drought stress, Table S7: Putative cis elements found in promoters of dehydrins in woody plants, Figure S1: An example of atypical architectures in woody dehydrins. (a) YSK_2_(KS)SK_3_Y_2_SK_3_ dehydrin from *Malus domestica.* (b) Y_2_(KY_3_)_3_Y_2_K_2_ dehydrin from *Juglans regia.* References [23,24,25,26,28,34,36,37,38,39,40,41,42,43,45,47,48,49,50,51,52,53,54,55,56,57,58,59] are cited in Supplementary Materials.

## References

[1] Amara I., Zaidi I., Masmoudi K., Ludevid M.D., Pages M., Goday A., Brini F. (2014). Insights into Late Embryogenesis Abundant (LEA) Proteins in Plants: From Structure to the Functions. Am. J. Plant Sci..

[2] Hernandez-Sanchez I.E., Lopez I.M., Martinez-Martinez C., Janis B., Jimenez-Bremont J.F., Covarrubias A.A., Menze M.A., Graether S.P., Thalhammer A. (2022). LEAfing through literature: Late embryogenesis abundant proteins coming of age-achievements and perspectives. J. Exp. Bot..

[3] Sun Z.P., Li S.Y., Chen W.Y., Zhang J.Q., Zhang L.X., Sun W., Wang Z.L. (2021). Plant Dehydrins: Expression, Regulatory Networks, and Protective Roles in Plants Challenged by Abiotic Stress. Int. J. Mol. Sci..

[4] Graether S.P., Boddington K.F. (2014). Disorder and function: A review of the dehydrin protein family. Front. Plant Sci..

[5] Smith M.A., Graether S.P. (2022). The Disordered Dehydrin and Its Role in Plant Protection: A Biochemical Perspective. Biomolecules.

[6] Malik A.A., Veltri M., Boddington K.F., Singh K.K., Graether S.P. (2017). Genome Analysis of Conserved Dehydrin Motifs in Vascular Plants. Front. Plant Sci..

[7] Close T.J. (1996). Dehydrins: Emergence of a biochemical role of a family of plant dehydration proteins. Physiol. Plant..

[8] Hara M. (2010). The multifunctionality of dehydrins: An overview. Plant Signal. Behav..

[9] Alsheikh M.K., Heyen B.J., Randall S.K. (2003). Ion binding properties of the dehydrin ERD14 are dependent upon phosphorylation. J. Biol. Chem..

[10] Goday A., Jensen A.B., Culianezmacia F.A., Alba M.M., Figueras M., Serratosa J., Torrent M., Pages M. (1994). The maize abscisic acid-responsive protein Rab17 is located in the nucleus and interacts with nuclear-localization signals. Plant Cell.

[11] Strimbeck G.R. (2017). Hiding in plain sight: The F segment and other conserved features of seed plant SKn dehydrins. Planta.

[12] Close T.J. (1997). Dehydrins: A commonality in the response of plants to dehydration and low temperature. Physiol. Plant..

[13] Kosova K., Vitamvas P., Prasil I.T. (2007). The role of dehydrins in plant response to cold. Biol. Plant..

[14] Berry Z.C., Avila-Lovera E., De Guzman M.E., O’Keefe K., Emery N.C. (2021). Beneath the Bark: Assessing Woody Stem Water and Carbon Fluxes and Its Prevalence Across Climates and the Woody Plant Phylogeny. Front. For. Glob. Chang..

[15] Azarkovich M.I. (2020). Dehydrins in Orthodox and Recalcitrant Seeds. Russ. J. Plant Physiol..

[16] Schneider T.D., Stephens R.M. (1990). Sequence logos—A new way to display consensus sequences. Nucleic Acids Res..

[17] Sievers F., Higgins D.G. (2020). QuanTest2: Benchmarking multiple sequence alignments using secondary structure prediction. Bioinformatics.

[18] Minh B.Q., Schmidt H.A., Chernomor O., Schrempf D., Woodhams M.D., von Haeseler A., Lanfear R. (2020). IQ-TREE 2: New Models and Efficient Methods for Phylogenetic Inference in the Genomic Era. Mol. Biol. Evol..

[19] Jones D.T., Taylor W.R., Thornton J.M. (1992). The rapid generation of mutation data matrices from protein sequences. Bioinformatics.

[20] Goodstein D.M., Shu S., Howson R., Neupane R., Hayes R.D., Fazo J., Mitros T., Dirks W., Hellsten U., Putnam N. (2012). Phytozome: A comparative platform for green plant genomics. Nucleic Acids Res..

[21] Lescot M., Déhais P., Thijs G., Marchal K., Moreau Y., Van de Peer Y., Rouzé P., Rombauts S. (2002). PlantCARE, a database of plant cis-acting regulatory elements and a portal to tools for in silico analysis of promoter sequences. Nucleic Acids Res..

[22] Svensson J., Ismail A.M., Palva E.T., Close T.J., Storey K.B., Storey J.M. (2002). Dehydrins. Cell and Molecular Response to Stress.

[23] Xiao H.G., Nassuth A. (2006). Stress- and development-induced expression of spliced and unspliced transcripts from two highly similar dehydrin 1 genes in V-riparia and V-vinifera. Plant Cell Rep..

[24] Cui H.W., Wang Y., Yu T.Q., Chen S.L., Chen Y.Z., Lu C.F. (2020). Heterologous Expression of Three *Ammopiptanthus mongolicus* Dehydrin Genes Confers Abiotic Stress Tolerance in *Arabidopsis thaliana*. Plants.

[25] Bae E.K., Lee H., Lee J.S., Noh E.W. (2009). Differential expression of a poplar SK2-type dehydrin gene in response to various stresses. BMB Rep..

[26] Zhang D., Yang T.C., Ren L. (2021). Y2SK2- and SK3-type dehydrins from *Agapanthus praecox* act as protectants to improve plant cell viability during cryopreservation. Plant Cell Tissue Organ Cult..

[27] Hara M., Endo T., Kamiya K., Kameyama A. (2017). The role of hydrophobic amino acids of K-segments in the cryoprotection of lactate dehydrogenase by dehydrins. J. Plant Physiol..

[28] Yokoyama T., Ohkubo T., Kamiya K., Hara M. (2020). Cryoprotective activity of *Arabidopsis* KS-type dehydrin depends on the hydrophobic amino acids of two active segments. Arch. Biochem. Biophys..

[29] Murray M.R., Graether S.P. (2022). Physiological, Structural, and Functional Insights Into the Cryoprotection of Membranes by the Dehydrins. Front. Plant Sci..

[30] Smith M.A., Graether S.P. (2022). The Effect of Positive Charge Distribution on the Cryoprotective Activity of Dehydrins. Biomolecules.

[31] Eriksson S.K., Kutzer M., Procek J., Grobner G., Harryson P. (2011). Tunable Membrane Binding of the Intrinsically Disordered Dehydrin Lti30, a Cold-Induced Plant Stress Protein. Plant Cell.

[32] Koag M.C., Wilkens S., Fenton R.D., Resnik J., Vo E., Close T.J. (2009). The K-Segment of Maize DHN1 Mediates Binding to Anionic Phospholipid Vesicles and Concomitant Structural Changes. Plant Physiol..

[33] Maszkowska J., Debski J., Kulik A., Kistowski M., Bucholc M., Lichocka M., Klimecka M., Sztatelman O., Szymanska K.P., Dadlez M. (2019). Phosphoproteomic analysis reveals that dehydrins ERD10 and ERD14 are phosphorylated by SNF1-related protein kinase 2.10 in response to osmotic stress. Plant Cell Environ..

[34] Hara M., Shinoda Y., Tanaka Y., Kuboi T. (2009). DNA binding of citrus dehydrin promoted by zinc ion. Plant Cell Environ..

[35] Boddington K.F., Graether S.P. (2019). Binding of a *Vitis riparia* dehydrin to DNA. Plant Sci..

[36] Hara M., Terashima S., Fukaya T., Kuboi T. (2003). Enhancement of cold tolerance and inhibition of lipid peroxidation by citrus dehydrin in transgenic tobacco. Planta.

[37] Hara M., Fujinaga M., Kuboi T. (2004). Radical scavenging activity and oxidative modification of citrus dehydrin. Plant Physiol. Biochem..

[38] Realini C., Rogers S.W., Rechsteiner M. (1994). KEKE motifs: Proposed roles in protein-protein association and presentation of peptides by MHC Class-I receptors. FEBS Lett..

[39] Ohkubo T., Kameyama A., Kamiya K., Kondo M., Hara M. (2020). F-segments of *Arabidopsis* dehydrins show cryoprotective activities for lactate dehydrogenase depending on the hydrophobic residues. Phytochemistry.

[40] Panchy N., Lehti-Shiu M., Shiu S.H. (2016). Evolution of Gene Duplication in Plants. Plant Physiol..

[41] Kleinwächter M., Radwan A., Hara M., Selmar D. (2014). Dehydrin expression in seeds: An issue of maturation drying. Front. Plant Sci..

[42] Sunderlikova V., Salaj J., Kopecky D., Salaj T., Wilhem E., Matusikova I. (2009). Dehydrin genes and their expression in recalcitrant oak (*Quercus robur*) embryos. Plant Cell Rep..

[43] Pacini E., Dolferus R. (2019). Pollen developmental arrest: Maintaining pollen fertility in a world with a changing climate. Front. Plant Sci..

[44] Firon N., Nepi M., Pacini E. (2012). Water status and associated processes mark critical stages in pollen development and functioning. Ann. Bot..

[45] Liang D., Xia H., Wu S., Ma F.W. (2012). Genome-wide identification and expression profiling of dehydrin gene family in *Malus domestica*. Mol. Biol. Rep..

[46] Zolotarov Y., Stromvik M. (2015). De Novo Regulatory Motif Discovery Identifies Significant Motifs in Promoters of Five Classes of Plant Dehydrin Genes. PLoS ONE.

[47] Jyothi-Prakash P.A., Mohanty B., Wijaya E., Lim T.M., Lin Q.S., Loh C.S., Kumar P.P. (2014). Identification of salt gland-associated genes and characterization of a dehydrin from the salt secretor mangrove *Avicennia officinalis*. BMC Plant Biol..

[48] Sadder M.T., Al-Doss A.A. (2014). Characterization of dehydrin AhDHN from Mediterranean saltbush (*Atriplex halimus*). Turk. J. Biol..

[49] Porat R., Pasentsis K., Rozentzvieg D., Gerasopoulos D., Falara V., Samach A., Lurie S., Kanellis A.K. (2004). Isolation of a dehydrin cDNA from orange and grapefruit citrus fruit that is specifically induced by the combination of heat followed by chilling temperatures. Physiol. Plant..

[50] Porat R., Pavoncello D., Lurie S., McCollum T.G. (2002). Identification of a grapefruit cDNA belonging to a unique class of citrus dehydrins and characterization of its expression patterns under temperature stress conditions. Physiol. Plant..

[51] Kalemba E.M., Pukacka S. (2008). Changes in late embryogenesis abundant proteins and a small heat shock protein during storage of beech (*Fagus sylvatica* L.) seeds. Environ. Exp. Bot..

[52] Deng Z.X., Wang Y.D., Jiang K.J., Liu X.F., Wu W.S., Gao S., Lin J., Sun X.F., Tang K.X. (2006). Molecular cloning and characterization of a novel dehydrin gene from *Ginkgo biloba*. Biosci. Rep..

[53] Omar S.A., Elsheery N.I., Kalaji H.M., Ebrahim M.K.H., Pietkiewicz S., Lee C.H., Allakhverdiev S.I., Xu Z.F. (2013). Identification and differential expression of two dehydrin cDNAs during maturation of *Jatropha curcas* seeds. Biochem. Mosc..

[54] Kjellsen T.D., Yakovlev I.A., Fossdal C.G., Strimbeck G.R. (2013). Dehydrin accumulation and extreme low-temperature tolerance in Siberian spruce (*Picea obovata*). Tree Physiol..

[55] Xu H.X., Yang Y., Xie L., Li X.Y., Feng C., Chen J.W., Xu C.J. (2014). Involvement of Multiple Types of Dehydrins in the Freezing Response in Loquat (*Eriobotrya japonica*). PLoS ONE.

[56] Vornam B., Gailing O., Derory J., Plomion C., Kremer A., Finkeldey R. (2011). Characterisation and natural variation of a dehydrin gene in Quercus petraea (Matt.) Liebl. Plant Biol..

[57] Fernandez-Caballero C., Rosales R., Romero I., Escribano M.I., Merodio C., Sanchez-Ballesta M.T. (2012). Unraveling the roles of CBF1, CBF4 and dehydrin 1 genes in the response of table grapes to high CO2 levels and low temperature. J. Plant Physiol..

[58] Yang Y.Z., He M.Y., Zhu Z.G., Li S.X., Xu Y., Zhang C.H., Singer S.D., Wang Y.J. (2012). Identification of the dehydrin gene family from grapevine species and analysis of their responsiveness to various forms of abiotic and biotic stress. BMC Plant Biol..

[59] Wisniewski M., Webb R., Balsamo R., Close T.J., Yu X.-M., Criffith M. (1999). Purification. Immunolocalization, cryoprotective, and antifreeze activity of PCA60: A dehydrin from peach (*Prunus persica*). Physiol. Plant..

