# Supplementary material for: Comparative Analysis of Dehydrins from Woody Plant Species
Source: Biomolecules. 2024 Feb 20;14(3):250. doi: 10.3390/biom14030250 (PMC10967807; doi:10.3390/biom14030250)
Supplement: Supplementary file 1 [file biomolecules-14-00250-s001.zip › Figure S1.pdf]

a A0A498KQG3.Malus.domestica

MANYGNTTAEKTTEEYFGNPTYNGSAGAGRTDEFGNAVQHGRTNTGYGTADPKTRDEGIV
GHHGGPAVLQRSGSSSSSSEDDGLGGRRKKGLKEKIKEKLPGGGNKDDQYSGGTQTTTPY
GGGTTYKVGEQRQEKLPGGGNKDDQYSGGTQTTTPYGGGTTYKVGEQHQEKGTTDKIKKK
LPGGNRDDQYSHDTSTTAAAYGGTGRTGEPQEKKNMMDKIKEKLPGGHAETDEYGNPLGG
ATTGAATGGYGAHDTAAATTGHDYGRKEHHGVTGALHRSGSSSSSSEDDGLGGRRKKGL
KQKTKEKLHGSTTTDTTYGTAGTTYPGGHQQEKGMMDKIKDKLPGTGHKDDPHYSTTTHA
TTTTTPSGGATYTEEHHEKKGIMDKIKEKLPGGNHYLQMAHLQNHQYGAPPTAACGNPTQ
GTDENSNPLVPQVDKYGNPLGHHGATTGIAPATTGEYGAHHTAVVTTGYAATTAGGHGTL
GHDYGRKEHHGVTGMLHRSASSSSSSSEDDGLGGRRKKKGLKQKINEKLPGSTTTDTAYG
TADPGGHHQEKGMMDKIEDKLPSTGHKDDPRYSHTTTSTTTPSGGGTAYMEEHHEKKGIM
DKIIEKLRGGHHKI

b A0A833UHG8.Juglans.regia

MAHFQNEYGAAHSTDAYGDVIRKDEYGNVIPTDENANPIRHSGTTLQGGQQQQRHDLST
EYDGQGGRRNKGLKEKITENIPGVGNNDHRNDTSSATTDAYGNVIRNDEYGNVPTDEYGN
QVRHSGTTLKGGQQQQQQRHDLSTEYDGQGGRGNKGLKEKLTENIPGVGNNDHRNNTSSV
TTDAYGNVIRKDEYGNVIPTDEHGNPIRHSGTTLQGGQQQQQQLHDLSTEYDGQGGGRNK
GLKEKLMENIPGVGNNDHRNNTSSATKDAYGNVIRKDEYGNVIPTDGHGNPIRHSGTTLQ
GGQQQQQQLHDLSTEYDGQSGGRNKGLKEKLTENMIPGVGNNDHRNNTSSATKDAYGNVI
RKDEYGNVIPKDEHGNPIRHSGTTLQGGQQQQQQRHDLSTEYDGQGGRRNRGLKEKVTE
NIPGVGNKDHRNDTSSATTKTPTTTAGGYGTGEQAQYHQHAEKKGVMEKIKEKVRRFV

Figure S1. An example of atypical architectures in woody dehydrins. (**a**) $YSK_2(KS)SK_3Y_2SK_3$ dehydrin from *Malus domestica.* (**b**) $Y_2(KY_3)_3Y_2K_2$ dehydrin from *Juglans regia.*
