# Supplementary material for: Comparative Analysis of Dehydrins from Woody Plant Species
Source: Biomolecules. 2024 Feb 20;14(3):250. doi: 10.3390/biom14030250 (PMC10967807; doi:10.3390/biom14030250)
Supplement: Supplementary file 1 [file biomolecules-14-00250-s001.zip › Table S6.pdf]

Table S6. Distribution in architectural types of dehydrins within plant species and their inducibility under drought stress.

Plant species	Total[a]	Architectural types[b]								Abiotic stress				Literature
		**Kn**	**KnS**	**Skn**	**YnKn**	**YnSKn**	**FnKn**	**FSKn**	**Atyp**	**Drought**	**Cold**	**Salinity**	**Heavy metals**	
Actinidia chinensis	2		2											
Actinidia deliciosa	7					2		5		AcDHN1	AcDHN1<br>AcDHN3<br>AcDHN7	AcDHN1<br>AcDHN5		[26]
Ammopiptanthus mongolicus	8		7		1						AmDHN154<br>AmDHN132<br>AmDHN200	AmDHN154		[39]
Ammopiptanthus nanus	1				1									
Avicennia marina	1				1									
Avicennia officinalis	2				2					AoDHN1		AoDHN1		[40]
Atriplex halimus	1							1				AhDHN		[41]
Atriplex canescens	1							1						
Citrus unshiu	2		2								CuCOR19		CuCOR15	[37], [38], [28]
Citrus sinensis	1							1			CsDHN			[54]
Citrus paradisi	2		1					1			COR15			[42]
Corylus heterophylla	3	1				2								
Corylus mandshurica	1					1								
Coffea canephora	6					5		1						
Elaeis guineensis var. Tenera	4			1		1		2						
Eucalyptus grandis	4					3	1							
Fagus sylvatica	6		1	1		1	2	1		FsDHN1				[43]

Plant species	Total[a]	Architectural types[b]								Abiotic stress				Literature
		**Kn**	**KnS**	**Skn**	**YnKn**	**YnSKn**	**FnKn**	**FSKn**	**Atyp**	**Drought**	**Cold**	**Salinity**	**Heavy metals**	
Ginkgo biloba	1						1			GbDHN		GbDHN		[44]
Gossypium anomalum	1							1						
Gossypium aridum	1							1						
Gossypium armourianum	1							1						
Gossypium barbadense	3							3						
Gossypium darwinii	3							3						
Gossypium davidsonii	2							2						
Gossypium harknessii	2							2						
Gossypium klotzschianum	2							2						
Gossypium lobatum	2							2						
Gossypium mustelinum	1							1						
Gossypium raimondii	5						1	4						
Gossypium tomentosum	5							5						
Gossypium schwendimanii	1							1						
Gossypium trilobum	1							1						
Jatropha curcas	3					2		1		JcDHN2<br>JcDHN1				[45]
Juglans regia	17		1		6	2		3	5					
Malus baccata	13	1		1	3	4		4						
Malus domestica	16	3		1	1	3	1	6	1	MdDHN4<br>MdDHN1	MdDHN4<br>MdDHN6			[36]

Plant species	Total[a]	Architectural types[b]								Abiotic stress				Literature
		Kn	KnS	Skn	YnKn	YnSKn	FnKn	FSKn	Atyp	Drought	Cold	Salinity	Heavy metals	
Malus prunifolia	8	1		2	1	3		1		MdDHN6	MdDHN3<br>MdDHN7<br>MdDHN8<br>MdDHN9			
Picea abies	6			1			1	4						
Picea obovata	2	1						1			PoDHN6<br>PoDHN2			[46]
Picea sitchensis	14		1				5	8						
Pinus densata	1							1						
Pinus nigra	1							1						
Pinus pinea	1							1						
Pinus sylvestris	3	2		1										
Pistacia vera	1							1						
Populus alba	5	1						4		PoDHN	PoDHN	PoDHN		[47]
Populus canadensis	1							1						
Populus davidiana	5	1	1					3						
Populus deltoides	2						1	1						
Populus glandulosa	1							1						
Populus maximowiczii	1							1						
Populus nigra	1							1						
Populus trichocarpa	2	1						1						
Prunus armeniaca	6				3	1		2						
Prunus avium	1							1						
Prunus dulcis	1				1									

Plant species	Total[a]	Architectural types[b]								Abiotic stress				Literature
		Kn	KnS	Skn	YnKn	YnSKn	FnKn	FSKn	Atyp	Drought	Cold	Salinity	Heavy metals	
Prunus persica	6	2			3	1								
Prunus yedoensis	3				1			2						
Pseudotsuga macrocarpa	1							1						
Pseudotsuga menziesii	2	1						1						
Rhaphiolepis bibas	7				2	2		3			RbDHN4<br>RbDHN3			[48]
Quercus ilex	1	1												
Quercus lobata	8	2	1	1	1	2	1							
Quercus petraea	2	2								QpDHN3	QpDHN3			[49]
Quercus robur	2	1				1				QrDHN1<br>QrDHN3				[34]
Vitis amurensis	1					1								
Vitis cinerea var. Helleri	1					1								
Vitis davidii	2					2								
Vitis labrusca x Vitis vinifera	1					1				VvDHN1a				[50]
Vitis piasezkii	1					1								
Vitis pseudoreticulata	1					1								
Vitis qinlingensis	1					1								
Vitis riparia	2					2				VrDHN1a				[51]
Vitis rotundifolia	1					1								
Vitis rupestris	2					2								
Vitis vinifera	9			1		6		2		VvDHN1a	VvDHN1			[51]
Vitis x champinii	1					1								
Vitis yeshanensis	4					3		1			VyDHN2			[52]

[a] Total number of sequences
[b] Number of sequences of given architectural types
