# Supplementary material for: Comparative Analysis of Dehydrins from Woody Plant Species
Source: Biomolecules. 2024 Feb 20;14(3):250. doi: 10.3390/biom14030250 (PMC10967807; doi:10.3390/biom14030250)
Supplement: Supplementary file 1 [file biomolecules-14-00250-s001.zip › Table S7 .pdf]

Table S7 Putative cis elements found in promoters of dehydrins in woody plants.

Motif name	sequence	Function
**Light response**		
G-box	CACGTG	cis-acting regulatory element involved in light responsiveness
GT1-motif	GGTTAA	light responsive element
I-box	GGATAAGGTG	part of a light responsive element
ACE	GACACGTATG GCGACGTACC	cis-acting element involved in light responsiveness
GATA-motif	GATAGGA	part of a light responsive element
TCT-motif	TCTTAC	part of a light responsive element
Box 4	ATTAAT	part of a conserved DNA module involved in light responsiveness
BoxII	TGGTAATAA	part of a light responsive element
ATCT-motif	AATCTAATCC	part of a conserved DNA module involved in light responsiveness
Sp1	GGGCGG	light responsive element
MRE	AACCTAA	MYB binding site involved in light responsiveness
AT1-motif	AATTATTTTTTATT	part of a light responsive module
TCCC-motif	TCTCCCT	part of a light responsive element
3-AF1 binding site	TAAGAGAGGAA	light responsive element
L-box	ATCCCACCTAC	part of a light responsive element
LAMP-element	CTTTATCA	part of a light responsive element
GA motif	ATAGATAA	part of a light responsive element
chs-CMA2a	TCACTTGA	part of a light responsive element
AE-box	AGAAACAA	part of a module for light response
Gap-box	CAAATGAA(A/G)A	part of a light responsive element
**Hormone response**		
ABRE	ACGTG	cis-acting element involved in the abscisic acid responsiveness
TATC-box	TATCCCA	cis-acting element involved in gibberellin-responsiveness
P-box	CCTTTTG	gibberellin-responsive element
GARE-motif	TCTGTTG	gibberellin-responsive element
CGTCA-motif	CGTCA	cis-acting regulatory element involved in the MeJA-responsiveness
TGACG-motif	TGACG	cis-acting regulatory element involved in the MeJA-responsiveness
SARE	TTCGACCATCTT	cis-acting element involved in salicylic acid responsiveness
AuxRR-core	GGTCCAT	cis-acting regulatory element involved in auxin responsiveness
TGA-element	AACGAC	auxin-responsive element
**Stress response**		
MBS	CAACTG	MYB binding site involved in drought-inducibility

DRE core	GCCGAC	a coupling element of ABRE in response to ABA
DRE	TACCGACAT	cis-acting element involved in dehydration, low-temp, salt stresses
TC-rich repeats		cis-acting element involved in defense and stress responsiveness
STRE	AGGGG	stress responsive element involved in a wide range of stresses
WUN-motif	AAATTTCTT	wound-responsive element
WRE3	CCACCT	Wounding and pathogen response element
as-1	TGACG	involved in transcriptional activation of several genes by auxin and/or salicylic acid
LTR	CCGAAA	cis-acting element involved in low-temperature responsiveness
MYB recognition site	CCGTTG	Responsive to water stress
W box	TTGACC	a binding site for WRKY TFs
MYB	CAACTG<br>TAACCA<br>CAACCA	drought, salt, and ABA responsive elements
MYC	CATGTG<br>CAATTG<br>TCTCTTA<br>CAATTG<br>CATGTG	drought, and ABA responsive elements
ARE	AAACCA	cis-acting regulatory element essential for the anaerobic induction
circadian	CAAAGATATC	cis-acting regulatory element involved in circadian control
**Development**		
GCN4_motif	TGAGTCA	cis-regulatory element involved in endosperm expression
RY-element	CATGCATG	cis-acting regulatory element involved in seed-specific regulation
CAT box	GCCACT	cis-acting regulatory element related to meristem expression
