# Supplementary material for: Comparative Analysis of Dehydrins from Woody Plant Species
Source: Biomolecules. 2024 Feb 20;14(3):250. doi: 10.3390/biom14030250 (PMC10967807; doi:10.3390/biom14030250)
Supplement: Supplementary file 1 [file biomolecules-14-00250-s001.zip › Tables S2-S5.pdf]

Table S2. Position-specific probability matrix of the K-segment

Position	Amino acid																			
	A	C	D	E	F	G	H	I	K	L	M	N	P	Q	R	S	T	V	W	Y
1	#	#	0.03	0.49		0.02	0.03		0.18				#	0.13	0.11			#		
2			#	0.09		#	0.01		0.81		#	0.04		0.03	0.01	#				
3	0.03			0.05		#			0.81		#		0.01	#	0.06		0.01	0.01		
4			#	#		0.94						#				0.04	#	#		
5	0.02	#			0.21			0.19	#	0.30	0.16		#			#	0.01	0.09		0.02
6	0.01			#	0.04	#		0.07	0.21	0.30	0.26				#		0.04	0.08		
7	#		0.37	0.52		0.06						#		0.02		#	#	#		
8									0.90		#			0.09	#					
9	0.04				#			0.76		0.03	#	0.01			#	#	0.02	0.12		
10				0.03	#			#	0.90		0.02	#		0.01	#		0.03			
11	0.01		0.22	0.75		#	#		#			#		0.01			#	#		
12						#		#	0.86		#	0.05		0.01	0.05	#				
13		#		#	0.01		#	0.27	#	0.64	#		#				0.02	0.04		
14				0.02		0.03	0.03			#			0.84		#	0.06	0.02			
15	0.03	0.01	#	#	#	0.91	#		#			#	#	#	#	0.01	#	#		

# - $0<p<0.01$

Table S3. Position-specific probability matrix of the Y-segment

Position	Amino acid																			
	A	C	D	E	F	G	H	I	K	L	M	N	P	Q	R	S	T	V	W	Y
1	#	#				#	#	0.01	0.14		#	0.02		0.06	0.06	0.01	0.65	0.03		#
2	#		0.96	#			#					0.02				#				#
3	0.14		0.03	0.62		0.02			#		#	#	0.06	0.11			#	#		
4		0.09			0.08	#	0.10			0.02		0.03		#	#	0.01	#			0.65
5	0.07		0.02	#		0.91										#		#		
6			0.02				#		0.02			0.92		#	#	0.03	0.01			0.15
7	0.02		#		#			0.02					0.59	0.01	0.03		0.01	0.30		
8	#		#	#	#	#		0.51		0.06	0.05	0.01				0.02	0.09	0.22		

# - 0<p<0.01

Table S4. Position-specific probability matrix of the S segment

Position	Amino acid																			
	A	C	D	E	F	G	H	I	K	L	M	N	P	Q	R	S	T	V	W	Y
1			#	0.01	0.02	0.03	0.03		#	0.87							0.01	0.01		
2	#			#			0.81		#			0.02	0.01	0.07	0.03	0.04				
3	#	#	#	#	#	0.05	0.04	#	#	0.02		#	#	0.02	0.86	#	#	#	#	#
4	#			0.02	#		0.06					#				0.84	0.08			
5	0.02		0.18	0.02		0.44	0.10		0.02			0.16			0.01	0.05	0.01			
6		#				0.04						0.01				0.95	#			
7			0.05			0.07						0.02			#	0.85	0.01			
8	#					0.02										0.98				
9	0.01												0.05			0.99	#			
10	#			0.09	#	0.03						#	#			0.86	#			
11			0.09	0.02	#	#										0.87	#			
12			0.13	0.02		#			#							0.82	#			#
13			0.37	0.22		0.11			#							0.25				
14			0.42	0.43		0.02	0.01					#	#	0.09		#				
15				0.64		0.12				0.02										

# - 0<p<0.01

Table S5. Position-specific probability matrix of the F-segment

Position	Amino acid																			
	A	C	D	E	F	G	H	I	K	L	M	N	P	Q	R	S	T	V	W	Y
1	0.80	0.02	0.02	0.07		0.04				#			0.03				0.02	#		
2	0.02			0.08		0.14			#		0.05		0.15	0.02			0.03	0.50		
3	0.05			0.94										#						
4	0.06		#			0.03	0.10	0.03	0.02			0.03		0.02	#	0.26	0.42	0.02		
5				0.02					0.59				#	0.22	0.03		0.15			
6			0.78	0.09		0.08						0.02			#	0.03				
7		0.08						#							0.92					
8				#		0.97										#				
9					#			0.04		0.66	0.28									#
10					0.98					0.02										
11	#		0.76			0.20						0.03					#			
12		#	#		0.81					0.16							#			
13					0.15	0.02	0.02			0.53	0.28									#
14			#			0.81			0.18	#										
15						#			0.97	#							0.02			

# - $0<p<0.01$
